# Whole conversion of agro-industrial wastes rich in galactose-based carbohydrates into lipid using oleaginous yeast *Aureobasidium namibiae*

**DOI:** 10.1186/s13068-021-02031-8

**Published:** 2021-09-15

**Authors:** Zhi-Peng Wang, Xin-Yue Zhang, Yan Ma, Jing-Run Ye, Jing Jiang, Hai-Ying Wang, Wei Chen

**Affiliations:** 1 School of Marine Science and Engineering, Qingdao Agricultural University, Qingdao, 266109 Shandong Province China; 2 School of Environmental Science and Engineering, Suzhou University of Science and Technology, Suzhou, 215009 Jiangsu Province China; 3 Key Laboratory of Sustainable Development of Polar Fishery, Ministry of Agriculture and Rural Affairs, Yellow Sea Fisheries Research Institute, Chinese Academy of Fishery Sciences, Qingdao, 266071 China

**Keywords:** Lipid, Soy molasses, Whey powder, α-Galactosidase, β-Galactosidase

## Abstract

**Background:**

Raw materials composed of easily assimilated monosaccharides have been employed as carbon source for production of microbial lipids. Nevertheless, agro-industrial wastes rich in galactose-based carbohydrates have not been introduced as feedstocks for oleaginous yeasts.

**Results:**

In this study, *Aureobasidium namibiae* A12 was found to efficiently accumulate lipid from soy molasses and whey powder containing galactose-based carbohydrates, with lipid productions of 5.30 g/L and 5.23 g/L, respectively. Over 80% of the fatty acids was C_16:0_, C_18:0_, C_18:1_, and C_18:2_. All kinds of single sugar components in the two byproducts were readily converted into lipids, with yields ranging between 0.116 g/g and 0.138 g/g. Three α-galactosidases and five β-galactosidases in the strain were cloned and analyzed. Changes of transcriptional levels indicated GalB and GalC were key α-galactosidases, and GalG was key β-galactosidase. In 10 L fermentor, lipid production from SM and WP achieved 6.45 g/L and 6.13 g/L, respectively. β-galactosidase was responsible for lactose hydrolysis; sucrase and α-galactosidase both contributed to the efficient hydrolysis of raffinose and stachyose in a cooperation manner.

**Conclusions:**

This is a new way to produce lipids from raw materials containing galactose-based carbohydrates. This finding revealed the significance of sucrase in the direct hydrolysis of galactose-based carbohydrates in raw materials for the first time and facilitated the understanding of the efficient utilization of galactose-based carbohydrates to manufacture lipid or other chemicals in bioprocess.

**Graphic abstract:**

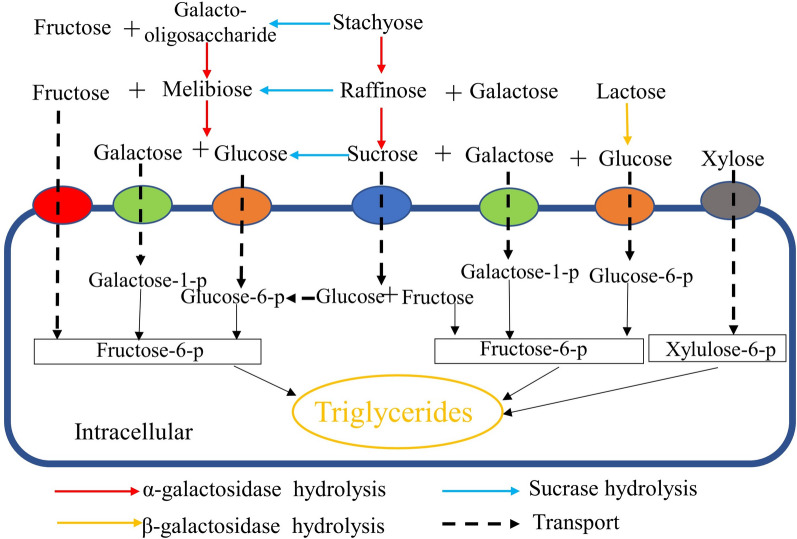

**Supplementary Information:**

The online version contains supplementary material available at 10.1186/s13068-021-02031-8.

## Background

Microbial lipid can be produced with high efficiency and has no competition from edible oil. Thus, microbial lipid has become important sources for producing biodiesel and other chemicals now [15, 39]. Among the lipid-producing microorganisms, oleaginous yeasts are attractive lipid producers for their rapid growth, large biomass, and high lipid yield [19, 33]. The reported oleaginous yeasts mainly belong to several genera, such as *Aureobasidium, Candida*, *Cryptococcus*, *Lipomyces*, *Rhodotorula*, *Rhodosporidium*, *Trichosporon*, and *Yarrowia* [8]. Among them, *Aureobasidium* *pullulans*, *Lipomyces starkeyi*, *Yarrowia lipolytica*, and *Rhodosporidium toruloides* can accumulate high level of neutral lipids, over 60% of their dry cell weight, from different feedstocks [8, 28, 31].

At present, the high production cost, especially the cost of carbon source for fermentation, impedes the industrial applications of microbial lipids [8]. In view of this, accumulating lipid from low-cost substrates is a feasible strategy to lower the production cost. A variety of raw materials or biowastes, including corncob, corn stover, wheat straw, cassava starch, waste oils, inulin, sugarcane bagasse, and sugarcane molasses, have been employed as carbon source for the lipid production by oleaginous yeasts [8]. Generally, raw materials can be converted into available monosaccharides using pretreatment methods, including acid hydrolysis, enzymatic hydrolysis, microbiological pretreatment, and detoxification process [8]. Corncob, corn stover, wheat straw, cassava starch, and sugarcane bagasse were converted into hydrolysate containing glucose as main component [8]. Inulin can be hydrolyzed into fructose [34].

Overall, the carbohydrates in these raw materials above mainly rely on glucose or fructose as monosaccharide unit, which can be easily assimilated. However, some agro-industrial by-products contain plenty of galactose-based carbohydrates, such as lactose and raffinose family oligosaccharides (RFOs) [35]. The galactoside bonds in the sugars are difficult to break up, thereby leading to limited applications and lowered commercial value. Due to lacking galactosidase synthesis and intracellular galactose assimilation pathway, most oleaginous yeasts cannot naturally consume galactose and RFO [35]. In addition, construction of galactose metabolic pathways in most yeast was not easy to achieve [18]. The abundant agro-industrial wastes containing galactose-based carbohydrates, such as whey powder (WP) and soy molasses (SM), have not been introduced as carbon source for lipid production [18, 35].

SM is a main by-product of the soy protein extraction from soybean. As soybean is one kind of staple crops providing plant proteins and lipid, SM is produced in large amount. Generally, SM has a high carbohydrate content of above 30% (w/v), mainly containing stachyose, raffinose, sucrose, monosaccharides, and other micronutrients [10, 22, 26]. Owing to the existence of α-(1,6) glycosidic bonds in RFO, SM cannot even be efficiently utilized by non-ruminant animals [35]. WP is a by-product in cheese production, and most of the production was concentrated in European and North America, with an annual worldwide production of about 165 million tons [16]. Despite whey containing many fermentable nutrients, such as lactose, proteins, and lipids, it has not efficiently utilized now [3]. In Europe, it was estimated that only 75% of the whey was recovered and developed as resources. While in the rest of the world, only than 50% of the whey was recovered [16].

*Aureobasidium* spp. has recently been evaluated as an excellent lipid producer and reported to efficiently assimilate various monosaccharides [31]. The genus was found to secrete many kinds of hydrolytic enzymes involved in biomass degradation [31]. In our previous study, several galactosidases were detected among extracellular substances of *Aureobasidium* sp., indicating the ability of assimilating galactose-based carbohydrates [41]. In this study, *Aureobasidium namibiae* strain A12 derived from mangrove was screened to accumulate high content of lipid directly from WP and SM.

## Results

### Evaluation of lipid producers of the yeast strains from SM

**Fig. 1:**
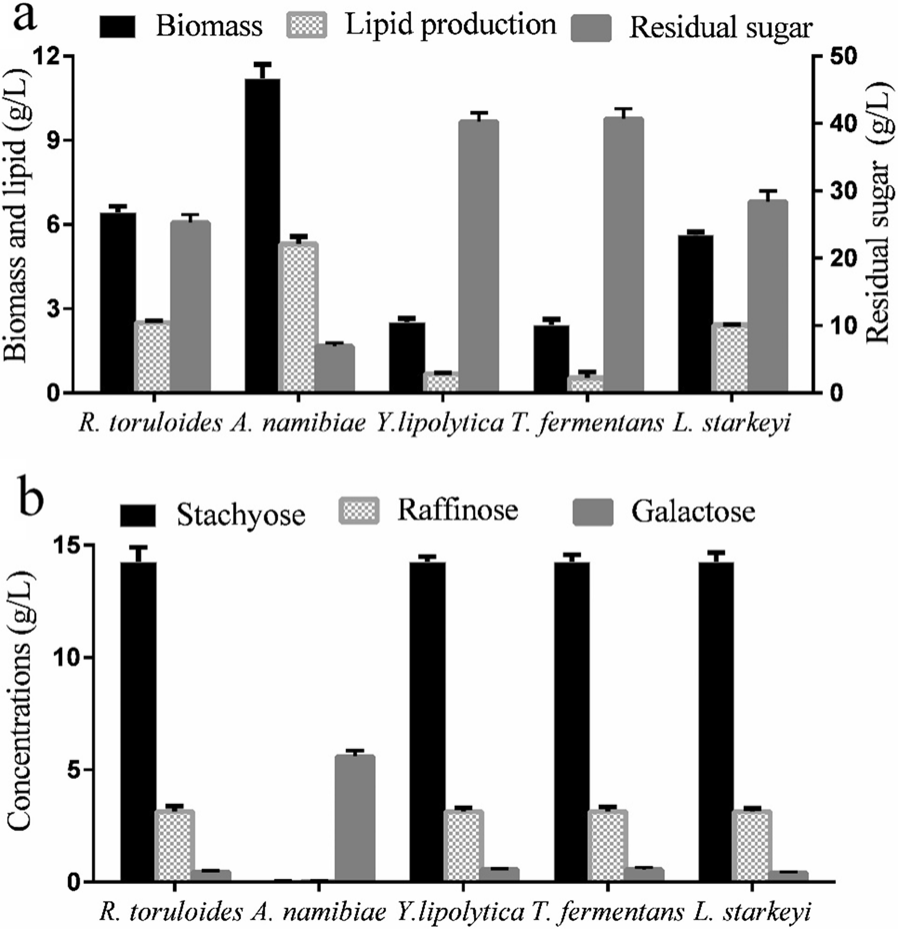
**a** Lipid productions of typical oleaginous yeasts and strain A12 from SM. **b** Contents of the residual galactose-based carbohydrates in the SM culture of typical oleaginous yeasts and strain A12. Data are given as means ± standard deviation, *n* = 3

Apart from raffinose and stachyose, SM contained high level of conventional sugars, such as glucose, fructose, and sucrose, accounting for more than 40% of total carbohydrates [35]. These conventional sugars provided enough carbon sources for lipid accumulation of *L. starkeyi* and *R. toruloides*, with the lipid production of 1.80 g/L and 1.93 g/L, respectively (Fig. 1a). The highest lipid content (47.3%, w/w) was observed in the strain A12 system, with the lipid production of 5.30 g/L and the biomass of 11.20 g/L (Fig. 1a). Obviously, only the conventional sugars in SM cannot support the large-scale lipid synthesis of strain A12. In the final culture of typical oleaginous yeasts, the galactose-based carbohydrates, including stachyose, raffinose, and galactose, in the culture of typical oleaginous yeasts remained the same with the original concentrations in the medium (Fig. 1b). Stachyose and raffinose was totally utilized by strain A12 (Fig. 1b). This indicated that the RFO should be converted into lipid in strain A12. The low content of residual sugar also provided another evidence for this.

### Evaluation of lipid producers of the yeast strains from WP

**Fig. 2:**
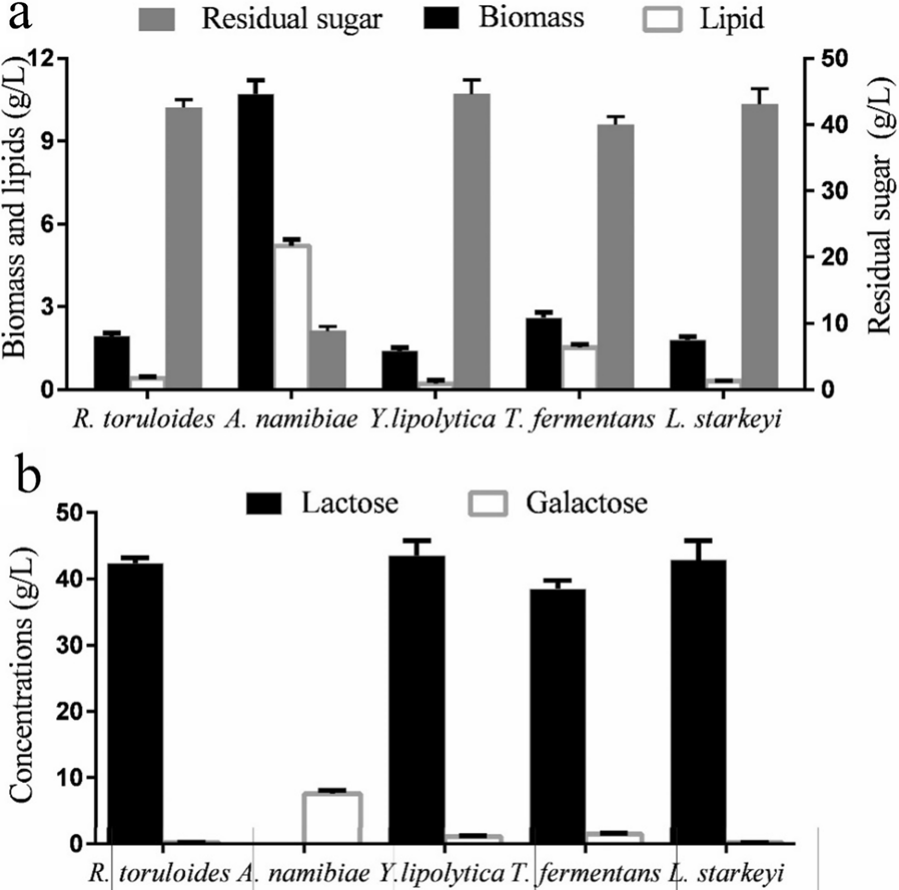
**a** Lipid productions of typical oleaginous yeasts and strain A12 from WP. **b** Contents of the residual galactose-based carbohydrates in the WP culture of typical oleaginous yeasts and strain A12. Data are given as means ± standard deviation, *n* = 3

Many researchers have made efforts to convert WP into citric acid, lactic acid, and lipases [2, 25, 36]. WP also has received a wide attention to serve as the raw material for the production of biofuels recently [18]. *A. namibiae* strain A12 and other four typical oleaginous yeasts were evaluated from the perspective of the capability to produce lipid from WP, whose main component is lactose. As shown in Fig. 2a, strain A12 possessed the highest lipid content (48.6%, w/w) and was able to accumulate 5.23 g/L lipid and 10.72 g/L biomass from WP. On the contrary, *L. starkeyi*, *Y. lipolytica*, and *R. toruloides* can only accumulate a small amount of biomass and lipid compared with strain A12. As shown in Fig. 2b, four typical oleaginous yeasts can just utilize little amount of the lactose. This indicates that strain A12 can utilize lactose as the main component in WP, while the other three oleaginous strains can only assimilate the monosaccharides with low concentrations as carbon source.

### Composition of the fatty acids

Triacylglycerols (TAGs), the reported main components of lipid in oleaginous yeasts, were stored in intracellular lipid droplets: One to three droplets in each cell of strain A12 (data not shown). After being stained by Nile Red, the lipid droplets presented yellow fluorescence which can be observed under a fluorescent microscope with blue excitation light. GC analysis of the fatty acids showed that the content of linoleic acid (C_18:2_), oleic acid (C_18:1_), stearic acid (C_18:0_), palmitoleic acid (C_16:1_), and palmitic acid (C_16:0_) in lipid converted from SM were 9.83%, 57.42%, 10.51%, 2.1%, and 20.14%, respectively (Table 1). Oleic acid (C_18:1_) and palmitic acid (C_16:0_) in lipid of *A. namibiae* converted from SM account for high proportion (78.94%). The composition of the fatty acids in lipid converted from glucose and WP was rather similar to that converted from SM.

**Table 1:** Composition of the fatty acids cultivated in different carbon sources

Fatty acid composition (%)	C_16:0_	C_16:1_	C_18:0_	C_18:1_	C_18:2_
Glucose	20.56 ±	3.10 ± 0.35	10.43 ± 0.16	58.38 ± 0.43	7.53 ± 0.27
SM	20.14 ± 0.43	2.10 ± 0.12	10.51 ± 0.61	57.42 ± 0.89	9.83 ± 0.42
WP	20.24 ± 0.11	2.62 ± 0.24	10.56 ± 0.26	57.89 ± 0.31	8.69 ± 0.23

### Analysis on the utilization of different sugars

**Fig. 3:**
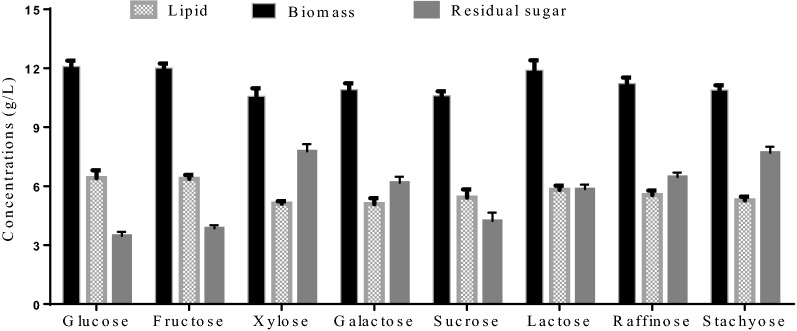
Lipid productions of strain A12 from single sugar components in SM and WP. Data are given as means ± standard deviation, *n* = 3

To further demonstrate the ability of strain A12 to utilize the waste sugars with galactoside bonds, it was cultured in a lipid-producing medium in which a single component in SM and WP was employed as the sole carbon source, including glucose, galactose, fructose, lactose, sucrose, xylose, stachyose, and raffinose. As shown in Fig. 3, the lipid production, biomass, and residual sugars in these fermentations have been compared. The test monosaccharides, which are present in SM and WP or exist as monosaccharide units of other sugars, were all readily converted to lipid. The lipid yield from them was not much different, ranging from 0.116 g/g to 0.138 g/g. This suggests that an efficient galactose and metabolism pathway indeed exists in the strain A12. It should be noted that this strain can even convert xylose into lipid, which is quite challenging for many other yeasts [8]. Besides, it can utilize the sucrose, commonly used as a conventional carbon source, and galactose-based sugars in an efficient manner, revealing the existence of a developed galactosidase system [6, 30].

### Galactosidase system in strain A12

**Fig. 4:**
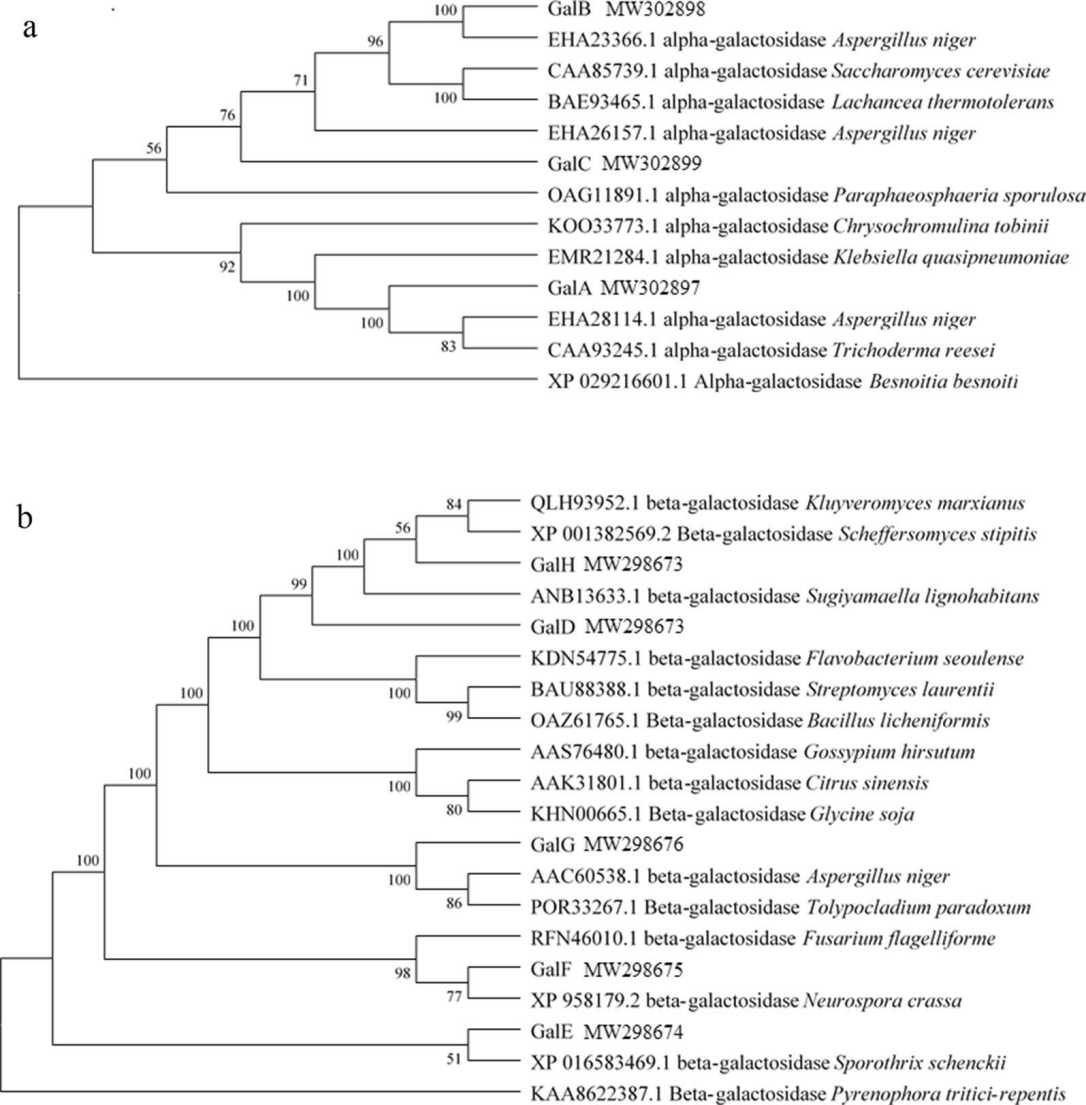
The phylogenetic tree generated with the neighbor-joining method based on α-galactosidase (**a**) and β-galactosidase sequences (**b**). Branch-related numbers are bootstrap values (confidence limits)

α-Galactosidase is effective in catalyzing the generation of α-linked galactose residues from various substrates, thus being crucial for the conversion of RFO into conventional carbon source sucrose, while β-galactosidase is able to cut the β-linked galactose residue of lactose to yield glucose [18, 35]. Based on the genome sequence of the type strain for this species, *A. namibiae* CBS 147.97, the potential galactosidase coding gene in strain A12 was cloned and analyzed. As listed in Table 2, diverse α-galactosidases and β-galactosidases were constituted by different numbers of amino acids. Through analysis, three potential α-galactosidases (namely GalA, GalB, and GalC) and five potential β-galactosidases (i.e., GalD, GalE, GalF, GalG, and GalH) were found. α-Galactosidases in strain A12 have been classified into two glycoside hydrolase families GH27 and GH36. GH27 and GH36 are perceived to have a common ancestral gene and hence they share a common catalytic mechanism. The phylogenic tree showed that GalB and GalC from GH27 are clustered into the same branch, while GalA belongs to another branch (Fig. 4). Each α-galactosidase of *A. namibiae* A12 is found very close to that of *Aspergillus niger*. This is in accordance with the recognition that the genus *Aureobasidium* is close to *A. niger* in terms of evolutionary relationship [21]. Nevertheless, β-galactosidases of *A. namibiae* A12 from two glycoside hydrolase families are much more diverse. Although the five β-galactosidases in *A. namibiae* A12 are all separated from β-galactosidases from plants and bacteria, they diverge in four branches close to specific basidiomycetes or ascomycetes, forming subclasses of specialized enzymes.

**Table 2:** Potential α-galactosidases, β-galactosidases in strain A12 and the basic characteristics

Proteins	Accession	Function	Super family	Amino acids	Signal peptide
GalA	MW302897	α-galactosidase	GH36	750	Yes
GalB	MW302898	α-galactosidase	GH27	452	Yes
GalC	MW302899	α-galactosidase	GH27	522	No
GalD	MW298673	β-galactosidase	GH2	858	No
GalE	MW298674	β-galactosidase	GH35	1001	Yes
GalF	MW298675	β-galactosidase	GH35	1002	Yes
GalG	MW298676	β-galactosidase	GH35	1009	Yes
GalH	MW298673	β-galactosidase	GH7	1071	No

**Fig. 5:**
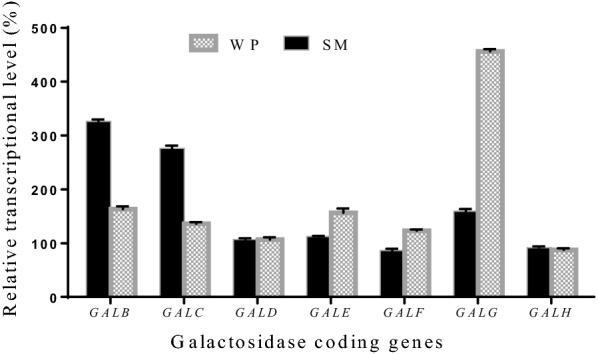
Changes of transcriptional levels of galactosidase coding genes

When *A. namibiae* A12 was cultivated in the LP medium containing SM, the transcriptional levels of *GALB* and *GALC* increased to 326.3% and 276.1% of those cultivated in the LP medium containing glucose (Fig. 5). The transcriptional levels of the genes coding β-galactosidases were not obviously changed (Fig. 5). *GALA* cannot be detected by qRT-PCR. When *A. namibiae* A12 was cultivated in the LP medium containing WP, the transcriptional levels of *GALG* increased to 456.3%. The results above indicated that GalB and GalC were key α-galactosidases for utilizing galactose-based carbohydrates in SM; GalG was key β-galactosidase for utilizing galactose-based carbohydrates in WP. To explore the characteristics of key galactosidases, GalB, GalC, and GalG were heterologously expressed. The molecular weights of GalB, GalC, and GalG were determined as 50.0 kDa, 55.3 kDa, and 107.2 kDa, respectively (Additional file 1: Fig. S1–S3). The molecular weights were consistent with those deduced from the amino acid sequences. Recombinant GalB and GalC can efficiently hydrolyze stachyose and raffinose (Additional file 1: Fig. S1, Fig. S2). Recombinant GalB peaked at 40 °C and was maintained higher than 60% at 30 °C–60 °C; GalB showed high activity and stability in a broad pH range (4.0–7.0) (Fig.S1). The characteristics of GalC were similar with those of GalB (Additional file 1: Fig. S1, S2). GalG showed higher in lower pH; the optimal pH for activity and stability was pH 4.0 (Additional file 1: Fig. S3). The three galactosidases can catalyze efficiently in the condition of lipid fermentation.

### Coordination of glycoside hydrolases during the lipid production

**Fig. 6:**
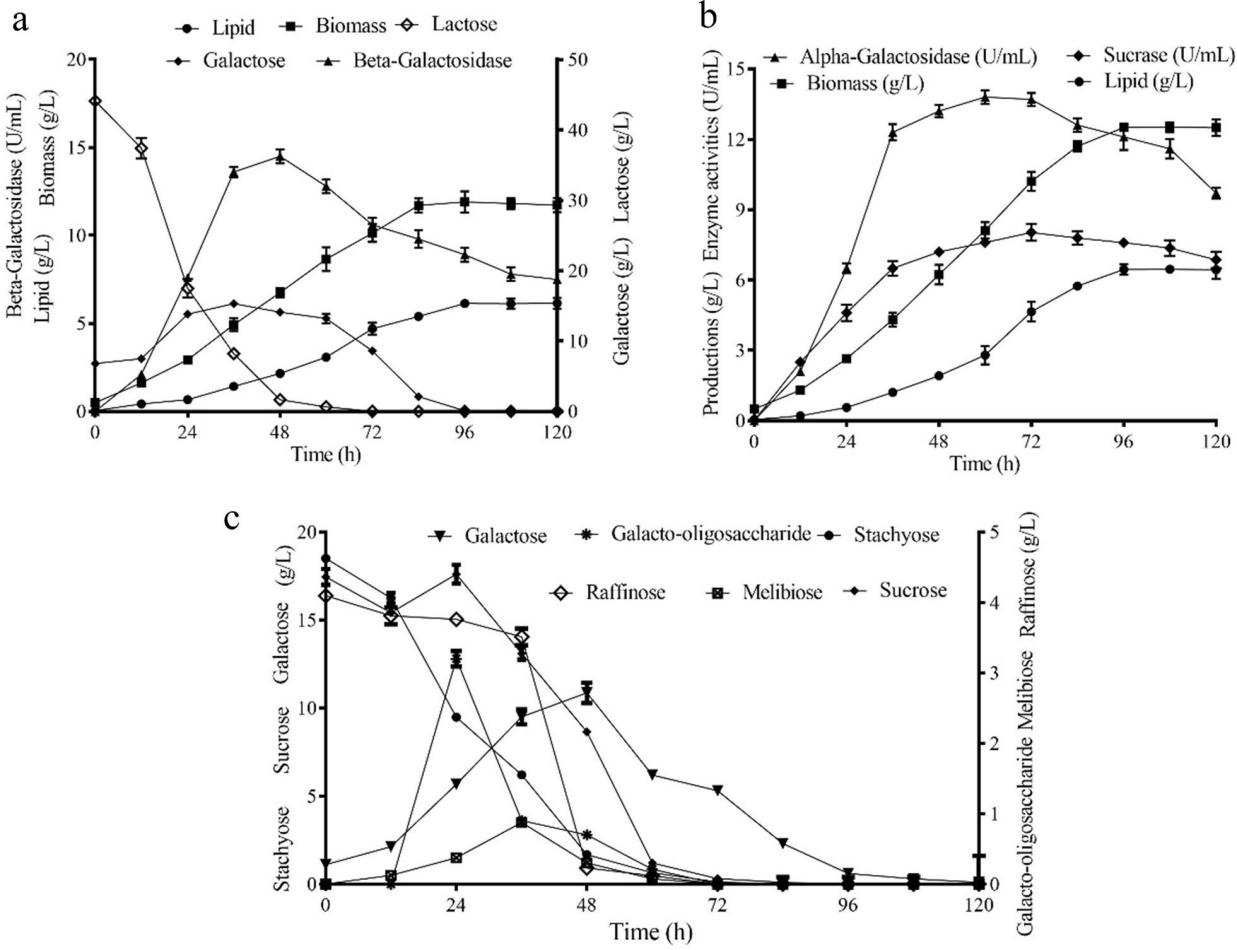
**a** Time course of lipid, biomass, galactosidases, and sugar contents in the 10L bioreactor during fermentation by strain A12 from WP. **b** Time course of lipid, biomass, sucrase and α-galactosidase in the 10 L bioreactor during fermentation by strain A12 from SM. **c** Time course of sugar contents during fermentation by strain A12 from SM. Data are given as means ± standard deviation, *n* = 3

The hydrolysis of carbohydrates in the medium can be realized by inducing microorganisms to generate corresponding glycoside hydrolases. To elucidate the role of glycoside hydrolases in the medium containing SM or WP as the carbon source, their activities and the concentrations of different sugars were monitored at a 12-h interval in a 10 L fermentor. The different activities of these glycoside hydrolases can be attributed to the different concentrations of the corresponding enzyme inducers, the substrates of the enzymes in the medium. As shown in Fig. 6a, β-galactosidase activity was 7.6 U/mL at 24 h in WP medium, while the activities of α-galactosidase and sucrase were at low levels of 0.6 U/mL and 1.3 U/mL, respectively. Galactose and glucose derived from lactose hydrolysis were then directed to lipid and biomass synthesis. After 72 h, no lactose was detected and the β-galactosidase started to decrease. At 96 h, galactose was completely consumed, with a maximal lipid production of 6.13 g/L. Adequate β-galactosidase enables the utilization of lactose in WP medium.

α-Galactosidase is responsible for converting raffinose family oligosaccharides into conventional sucrose and galactose. As shown in Fig. 6b and c, α-galactosidases and sucrase were induced much more than those in WP, with 22.6 g/L RFO detected. At 24 h, the activity of α-galactosidase reached 6.52 U/mL, with reduced content of stachyose and increased galactose; the activity of sucrase reached 4.60 U/mL. However, the content of raffinose just decreased slightly to 3.51 g/L at 36 h, as a result of the supplement through the release of raffinose from stachyose cut by α-galactosidase (Fig. 6b, c). Besides, the content of sucrose even increased to the level before 24 h. This indicated that the hydrolysis of sucrose by sucrase was slower than its generation from stachyose and raffinose catalyzed by α-galactosidase. At 48 h, the activity of α-galactosidase and sucrase reached 13.2 U/mL and 7.2 U/mL, respectively. Under the reaction of α-galactosidase and sucrase, sucrose and RFO were both not detected after 96 h, and the lipid achieved a maximal yield of 6.45 g/L (Fig. 6b, c).

## Discussion

As far as we know, this is the first time for the introduction of SM as low-cost carbon source in lipid production [8, 10, 22, 26]. Benefiting from the low cost and easy availability, SM has become a promising feedstock in China [35]. Moreover, the high carbohydrate content makes SM attractive among researchers and biorefinery industrialists [8, 10, 22, 26]. However, the current SM-derived fermentations suffer from limited efficiency, as most of them still focused on the utilization of the conventional constituents [7, 10, 27, 37]. Up to now, only the synthesis of poly-hydroxyalkanoate, propionic acid, and polymalic acid from RFO in SM was achieved [7, 12, 37].

The tested typical oleaginous yeasts in this study all have been proved capable of accumulating high level of lipid from various carbohydrates with glucose or fructose as the monosaccharide unit. *R. toruloides* accumulates lipid from cassava starch [20]. Inulin, sucrose, and tuber meal of Jerusalem artichoke have been used for lipid production in *Aureobasidium pullulans* [31]. The lipid content and lipid production of *Y. lipolytica* Po1g grown in sugarcane bagasse hydrolysate were 58.5% and 6.68 g/L, respectively [32]. Moreover, *R. toruloides*, *L. starkeyi*, and *Y. lipolytica* can produce lipid with the hydrolysate derived from the pretreatment of wheat straw [38]. As for galactose-based carbohydrates, only *A. namibiae* among the oleaginous yeasts can convert them into lipid. The composition of the fatty acids in lipid produced by *A. namibiae* was similar to that of plant oils [8]. C_18:1_ was also the predominant fatty acid in lipid of *R. toruloides*, *L. starkeyi,* and *Y. lipolytica*, with a content of more than 40% [8, 28, 31]. As revealed in previous studies, the long-chain fatty acids constituted by 16 and 18 carbon atoms were ideal candidates for biodiesel production via methylation.

Generally, very few yeasts possess galactosidases and galactose metabolism pathway, thus resulting in the impossibility to utilize lactose by most yeasts [9]. Actually, *A. namibiae* was the first oleaginous microorganism reported to produce lipid using SM as the feedstock. In previous studies, only two oleaginous yeasts have been reported able to accumulate lipid from WP with a content of more than 40% [6, 30]. *Cystobasidium oligophagum* has been characterized to efficiently convert WP into lipid. The biomass and the lipid content were 43.1% and 6.38 g/L, respectively [30]. In another study, the biomass and lipid production of *Cryptococcus curvatus* from WP achieved 10.77 g/L and 63.4%, respectively [6]. Moreover, a secreted β-galactosidase was introduced in *Y. lipolytica*, along with the enhanced cellular galactose metabolism. The engineered strain could achieve the rapid conversion of acid whey, producing 6.61 g/L of fatty acids [18]. In 10 L fermentor, lipid production by *A. namibiae* in the medium containing SM or WP as the carbon source achieved 6.45g/L and 6.13 g/L, respectively, with lipid content of 53.3% and 51.5%, respectively (Fig.6a, b). As shown in Fig. 6a and b, lipid production increased slowly than biomass accumulation in the first 48 h, but lipid production increased significantly in the 48–72 h. This indicated the lipid yield enhanced after 48-h culture. Due to the slow growth of the yeast cell, more carbon source was partitioned into lipid synthesis. The similar trend was also be confirmed in the lipid production of *R. toruloides* [20, 34].

The lipid productivity of *A. namibiae* A12 was slightly lower than that of *C. curvatus* strain, and much higher than that of *C. oligophagum* strain*,* using WP as the carbon source (Table 3). However, the lipid productivity of engineered *Y. lipolytica* strain achieved 0.092 g/L/h, remarkably higher than those of other strains. Lipid synthesis flux was significantly enhanced by overexpressing acetyl-CoA carboxylase and diacylglycerol acyltransferase in the engineered strains, thus caused a shorter fermentation period and higher lipid production [3]. The engineering strategy in *Y. lipolytica* can be adopted in *A. namibiae* A12 to optimize lipid production. And fed-batch fermentation will be another method to enhance the lipid production.

**Table 3:** Comparison of lipid productions by different strains from galactose-based carbohydrates

Strains	Feedstocks	Lipid content (%)	Productivity (g/L/h)	Ref.
*C. oligophagum*	Whey	44.12	0.034	[30]
*C. curvatus*	Whey	63	0.071	[6]
*Y. lipolytica*	Whey	about 45.5	0.092	[18]
*A. namibiae*	SM	53.3	0.067	This study
*A. namibiae*	WP	51.5	0.064	This study

Generally, similar compositions were found in the composition of fatty acids in the majority of oleaginous yeasts, including *Y. lipolytica*, *L. starkeyi*, and strain A12 [8, 28]. These results confirmed that SM and WP would be the ideal substrates for lipid production. Although the fermentations in SM and WP were probably slowed down due to the hydrolysis of raffinose and stachyose, lower osmotic pressures were allowed and the biomass was increased. Thus, as largely untapped resources currently, SM and WP deserve arousing attention from *A. namibiae* system to be a self-sufficient nutrient for numerous potential bioprocesses [8]. This galactosidases system provides molecular basis for strain A12 to utilize sugars with galactoside bonds. Based on the excellent the characteristics of GalB, GalC, and GalG (Additional file 1: Fig. S1; Fig. S2; Fig. S3), the three key galactosidases in *Aureobasidium* can be a valuable untapped industrial enzyme resource [21]. Besides, the sucrose-utilizing capacity has been proved among *Aureobasidium* strains to accumulate biopolymer and other products [1, 13, 24, 37]. In the genome of *Aureobasidium namibiae* CBS 147.97, ten potential sucrase coding genes have been identified, giving support for the capacity of *A. namibiae* A12 to utilize sucrose in SM.

**Fig. 7:**
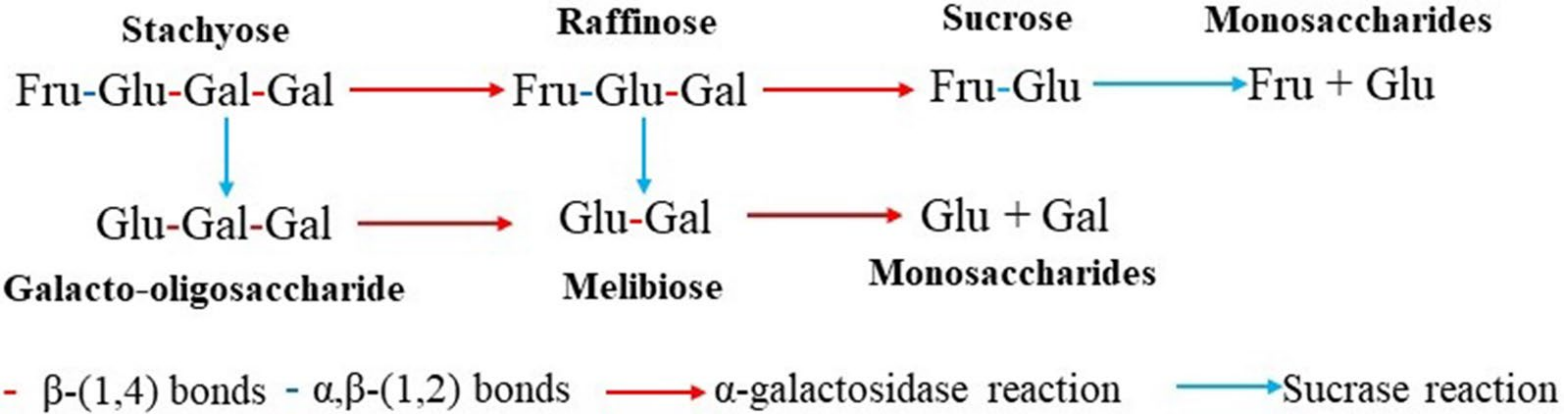
The contribution of sucrase and α-galactosidase to the hydrolysis of RFO. Glu: Glucose; Fuc: Fructose; Gal: Galactose

Unexpectedly, two kinds of sugars not present in SM medium have been detected during the fermentation, which were identified as melibiose and galacto-oligosaccharide. Their existence revealed the neglected fact that the sucrase also functions in the hydrolysis of stachyose and raffinose apart from α-galactosidase [29]. Sucrase has been proved to catalyze the release of fructose residues from different substrates, including stachyose and raffinose. Thus, sucrase and α-galactosidase both contribute to efficient hydrolysis of RFO in a cooperation manner, rather than in a sequential way [8, 29]. The sequence of cutting the two kinds of glycosidic bonds influences the generation of intermediate sugars, as shown in Fig. 7. In the case of sole α-galactosidase, RFO was converted into sucrose with the release of galactose; when only sucrose exists, melibiose and galacto-oligosaccharide were generated with the release of fructose (Fig. 7). This finding revealed the significance of sucrase in the direct hydrolysis of RFO in raw materials for the first time and facilitated the understanding of the efficient utilization of RFO to manufacture lipid or other chemicals in bioprocess [8, 35].

## Conclusions

*A. namibiae* A12 efficiently accumulate lipid from galactose-based SM and WP. β-Galactosidase was responsible for lactose hydrolysis in WP; sucrase and α-galactosidase both contributed to the efficient hydrolysis of RFO in a cooperation manner. These results demonstrate the feasibility of using SM and WP as a previously untapped resource for biotechnology. This platform strain can be further improved to produce high-value lipid-derived chemicals from waste sugars with galactoside bonds.

## Methods

### Strains and media

Four typical oleaginous yeast strains, i.e., *R. toruloides* R2, *Trichosporon fermentans* T1*, Y. lipolytica* URA, and *L. starkeyi* AM, were preserved at − 80 ℃ in our laboratory. Strain A12 was isolated from a plant in Zhanjiang Mangrove National Nature Reserve, and identified as *A. namibiae*. Yeast strains were cultivated in YPD medium (containing 20.0 g/L glucose, 20.0 g/L peptone, and 10.0 g/L yeast extract) [17]. The composition of lipid production (LP) medium included 50 g/L glucose, 3.0 g/L K_2_HPO_4_, 2.0 g/L KH_2_PO_4_, 0.1 g/L MgSO_4_·7H_2_O, and 2 g/L corn steep liquor (CSL), with pH 6.0. When other pure carbohydrates were selected as the carbon source, the concentrations of the carbohydrates remained 50 g/L. GPPB medium was used for SIase production and contained 30.0 g/L glucose, 1.0 g/L (NH4)_2_SO_4_, 6.0 g/L yeast extract, 2.0 g/L KH_2_PO_4_, 3.0 g/L K_2_HPO_4_, and 0.1 g/L MgSO_4_·7H_2_O with a pH of 6.0 [42].

### Pretreatment of SM

SM was adjusted to pH 10.0 with Ca(OH)_2_ powder which was held for 1 h. Subsequently, the liquid was filtered to remove excess Ca(OH)_2_ and adjusted to pH 6.0 with H_2_SO_4_, which was kept for 1 h. After 10 min of boiling, the solution was centrifuged at 7000 × *g*, followed by being stored at 4 °C [41]. The pretreated SM contained 3.32% (*w*/*w*) glucose, 2.81% (*w*/*w*) fructose, 13.42% (*w*/*w*) sucrose, 1.73% (*w*/*w*) xylose, 3.15% (*w*/*w*) raffinose, 14.26% (*w*/*w*) stachyose, and 0.78% (*w*/*w*) galactose.

### Lipid production at flask level

Strain A12 was cultivated in 5 mL YPD medium at 30 ℃ for 20 h which was then switched to 50 mL LP medium at the same temperature. The fermentation proceeded for a total of 120 h, after which the broth was centrifuged at 5000 × *g*. The yeast cells were harvested by centrifugation at 5000 × *g*, washed with sterile water, and dried at 80 ℃ overnight for the biomass detection. Nile Red (0.5 mg/L in DMSO, GenMed Scientifics Inc., USA) was adopted to stain the washed cells (10 μL) which were then observed under an Olympus U-LH100HG fluorescent microscope with blue excitation light [31]. In the experiments checking the lipid production from the wastes, glucose in LP medium was absent; SM and low protein WP concentrations were set at 130 g/L and 60 g/L, respectively. So that the sugar content in the medium can be maintained at about 50 g/L. The initial lactose and galactose in LP medium containing WP were maintained as 44.1 g/L and 6.8 g/L, respectively. The initial LP medium containing SM contained 4.3 g/L glucose, 3.7 g/L fructose, 17.5 g/L sucrose, 2.2 g/L xylose, 4.1 g/L raffinose, 18.5 g/L stachyose, and 1.0 g/L galactose, respectively.

### Molecular manipulations

The extraction of genomic DNA from the *A. namibiae* strain was conducted with the TIANamp Yeast DNA Kit (TIANGEN BIOTECH, China). The DNA polymerase was utilized according to the recommendation by the manufacturer (New England BioLabs, USA). The transformation of *E. coli* was realized by the heat shock method of Sambrook et al. [23]. The primers were designed based on the potential gene encoding galactosidase in *A. namibiae* CBS 147.97, and this potential gene in strain A12 was subjected to PCR amplification. The amplified DNA fragments were then transformed into *E. coli* DH5α (TaKaRa Biotechnology, China). The *E. coli* transformants were screened on LB agar which contained 100.0 μg/mL ampicillin and play a role in the plasmid amplification and DNA sequencing.

### Bioinformatics analysis of galactosidases

The gene protein sequence was compared using HMMER3 based on the CAZy (Carbohydrate-Active enZYmes) database. In this way, the annotation information of carbohydrate-active enzymes became available [4]. The genes coding for galactosidases and sucrases were amplified using primers listed in Additional file 1: Table S1. E-value < 1e-5 was adopted as the filter condition. SignalP 4.1 server (http://www.cbs.dtu.dk/services/SignalP-4.1/) was applied to the signal peptide analysis. The phylogenetic tree was constructed on the basis of reported α-galactosidase and β-galactosidase sequences by virtue of the neighbor-joining method in MEGA version 7.0.

### Gene expression level analyses with qRT-PCR assays

Strain A12 was inoculated for 36 h at 30 °C in the LP medium containing glucose, SM, and WP, respectively. Total RNA was isolated using TRIzol reagents described by the manufacturer. RNA concentration was analyzed with NanoDrop2000c spectrophotometer (Thermo Fisher, Germany) and reversed transcribed into cDNA. Real-time PCR was performed in triplicate using a SYBR green assay kit (Toyobo, Japan), with the SYBR real-time PCR master mix (Applied Biosystems). The primers for transcription-quantitative PCR (qRT-PCR) were designed according to the sequences of the genes coding galactosidases; 18S rDNA gene was employed as an internal reference (Additional file 1: Table S2). Relative gene expression level changes in LP medium containing SM and WP were analyzed by using the comparative CT method with a 10 μL reaction system. Samples from LP medium containing glucose was used as control. All of the amplifications were performed in triplicate from biological triplicates.

### Secretory expression of galactosidases

After codons were optimized, the genes coding galactosidases containing the XPR2 signal peptide gene was synthesized in Synbio Technologies, China. The DNA fragment synthesized in this study was transformed into the URA^−^ strain [42]. After the cultivation in GPPB liquid medium at 30 °C for 48 h, the positive transformants were detected for their galactosidase activities. The supernatant of positive transformant was adjusted to pH 7.5 and then loaded on gel filtration chromatography column as well as anion exchange chromatography column (GE Healthcare, USA). The Mw and purity of recombinant galactosidases were verified based on SDS-PAGE on 12% (w/v) gel.

### Effects of temperature and pH on activity and stability of recombinant galactosidases

The enzymatic reaction mediated by recombinant galactosidases proceeded in 10 mM glycine–NaOH buffer (pH 6.0) at temperatures ranging from 10 °C to 70 °C, the results of which showed the optimal reaction temperature. To investigate the thermal stability of recombinant galactosidases, the enzyme after purification was first incubated at temperatures ranging from 10 °C to 70 °C for 12 h and then the remaining activity at 40 °C was detected. Tannic acid solutions were prepared with 10 mM buffer at different pH levels (Na_2_HPO_4_–citric acid, pH 2.0–8.0; glycine–NaOH, pH 8.5–0.0) to act as the substrate for the determination of the optimal reaction pH. pH stability was estimated according to the remaining activity after the incubation at 4 °C for 12 h in buffer at different pH levels. All reactions were allowed to proceed in triplicate.

### Determination of fatty acid composition of the extracted oil

The cellular lipids were extracted and weighed with the method of Folch et al. [11]. For the sake of determining fatty acid composition, the oil obtained by Soxhlet extraction was directly subjected to transmethylation and fatty acid esters were then analyzed using gas chromatography (GC) according to the literature procedure.

### Enzymatic activity assay and sugar concentration determination

The α-galactosidase activity was evaluated with pNPG as substrate [5]. The reaction was terminated by Na_2_CO_3_ and the solution was filtered through a 0.22 μm membrane, followed by high-performance liquid chromatography (HPLC) analysis. The carbohydrate concentration was calculated depending on the retention time and peak area. One unit of the α-galactosidase activity (U) was defined as the amount of the enzyme that generated 1 μM *p*-nitrophenol per minute at 40 °C in buffer at pH 4.5.

The detection of β-galactosidase activity was carried out by the similar method [14]. The mixture of the culture (0.5 mL) and *o*-nitrophenyl-β-D-galactopyranoside (ONPG, 20.0 mM, 0.5 mL) in 100 mM citrate buffer (pH 4.0) underwent the incubation at 40 °C for 10 min. The β-galactosidase in the mixture was inactivated by 2.0 mL of 0.5 M Na_2_CO_3_ solution. One unit of the β-galactosidase activity (U) was defined as the amount of the enzyme producing 1.0 μM ONP per minute in the conditions of this study.

The sucrase activity was detected as reported [40]. The carbohydrate content was determined on an Agilent 1200 system (Agilent Technologies, USA) with amino (NH_2_) column (Thermo Scientific, USA).

### Lipid production in 10 L fermentor

Large-scale fermentation was performed in Biostat B 10 L fermentors (B. Braun, Germany) for the lipid production from WP and SM, respectively. Strain A12 was first inoculated in YPD medium (600 mL) as a seed culture and then transferred to the fermentor which contained 6 L of lipid production medium. In the next step, the fermentation began and lasted for 120 h under the following conditions: agitation speed: 300 rpm, aeration rate: 50 L/min, temperature: 30 ℃, and pH 6.0. The samples were also taken every 12 h to determine the biomass, lipid yield, and activities of α-galactosidase, β-galactosidase, and sucrase. Besides, the sugar content was monitored as well.

## Supplementary Information

**Additional file 1**: **Figure S1.** (a) SDS-PAGE analysis of recombinant GalB. Lane M, standard Mw markers; lane 1, purified GalB; lane 2, Bovine albumin. (b) Substrate specificity of GalB (c) The pH activity and stability of GalB. (d) Thetemperature activity and stability of GalB. **Figure S2.** (a) SDS-PAGE analysis of recombinant GalC. Lane M, standard Mw markers; lane 1, purified GalC; lane 2, Bovine albumin. (b) Substrate specificity of GalB (c) The pH activity and stability of GalC. (d) Thetemperature activity and stability of GalC. **Figure S3.** (a) SDS-PAGE analysis of recombinant GalG. Lane M, standard Mw markers; lane 1, purified GalG; lane 2, Bovine albumin. (b) The pH activity and stability of GalG. (c) Thetemperature activity and stability of GalG. **Table S1.** Primers for amplifying genes coding galactosidases. **Table S2.** Primers used to perform qRT-PCR assays.
