# Supplementary material for: Whole conversion of agro-industrial wastes rich in galactose-based carbohydrates into lipid using oleaginous yeast Aureobasidium namibiae
Source: Biotechnol Biofuels. 2021 Sep 15;14:181. doi: 10.1186/s13068-021-02031-8 (PMC8442318; doi:10.1186/s13068-021-02031-8)
Supplement: Supplementary file 1 — Additional file 1: Figure S1. (a) SDS-PAGE analysis of recombinant GalB. Lane M, standard Mw markers; lane 1, purified GalB; lane 2, Bovine albumin. (b) Substrate specificity of GalB (c) The pH activity and stability of GalB. (d) Thetemperature activity and stability of GalB. Figure S2. (a) SDS-PAGE analysis of recombinant GalC. Lane M, standard Mw markers; lane 1, purified GalC; lane 2, Bovine albumin. (b) Substrate specificity of GalB (c) The pH activity and stability of GalC. (d) Thetemperature activity and stability of GalC. Figure S3. (a) SDS-PAGE analysis of recombinant GalG. Lane M, standard Mw markers; lane 1, purified GalG; lane 2, Bovine albumin. (b) The pH activity and stability of GalG. (c) Thetemperature activity and stability of GalG. Table S1. Primers for amplifying genes coding galactosidases. Table S2. Primers used to perform qRT-PCR assays. [file 13068_2021_2031_MOESM1_ESM.docx]

**Whole conversion of agro-industrial wastes rich in galactose-based carbohydrates into lipid using oleaginous yeast *Aureobasidium namibiae***

Zhi-Peng Wang ^a,^ *, Xin-Yue Zhang ^a^, Yan Ma ^a^, Jing-Run Ye ^a^, Yan Ma ^a^, Jing Jiang ^b^, Hai-Ying Wang ^c^, Wei Chen ^a,^ *****

^a^ School of Marine Science and Engineering, Qingdao Agricultural University, Qingdao, Shandong Province, 266109, China

^b^ School of Environmental Science and Engineering, Suzhou University of Science and Technology, Suzhou, Jiangsu Province, 215009, China

^c^ Key Laboratory of Sustainable Development of Polar Fishery, Ministry of Agriculture and Rural Aﬀairs, Yellow Sea Fisheries Research Institute, Chinese Academy of Fishery Sciences, Qingdao, 266071, China

*Authors to whom any correspondence should be addressed

Emails: [wangzpmbio@163.com](mailto:wangzpmbio@163.com) (Zhi-Peng Wang); [chenwei@qau.edu.cn](mailto:chenwei@qau.edu.cn) (Wei Chen)


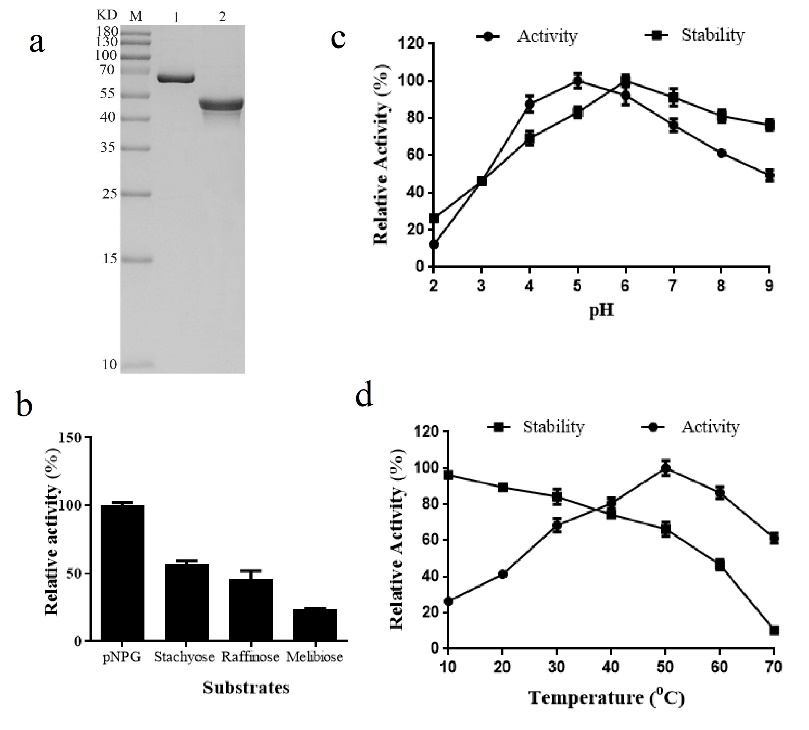


Figure S1. (a) SDS-PAGE analysis of recombinant GalB. Lane M, standard Mw markers; lane 1, purified GalB; lane 2, Bovine albumin. (b) Substrate specificity of GalB (c) The pH activity and stability of GalB. (d) Thetemperature activity and stability of GalB.


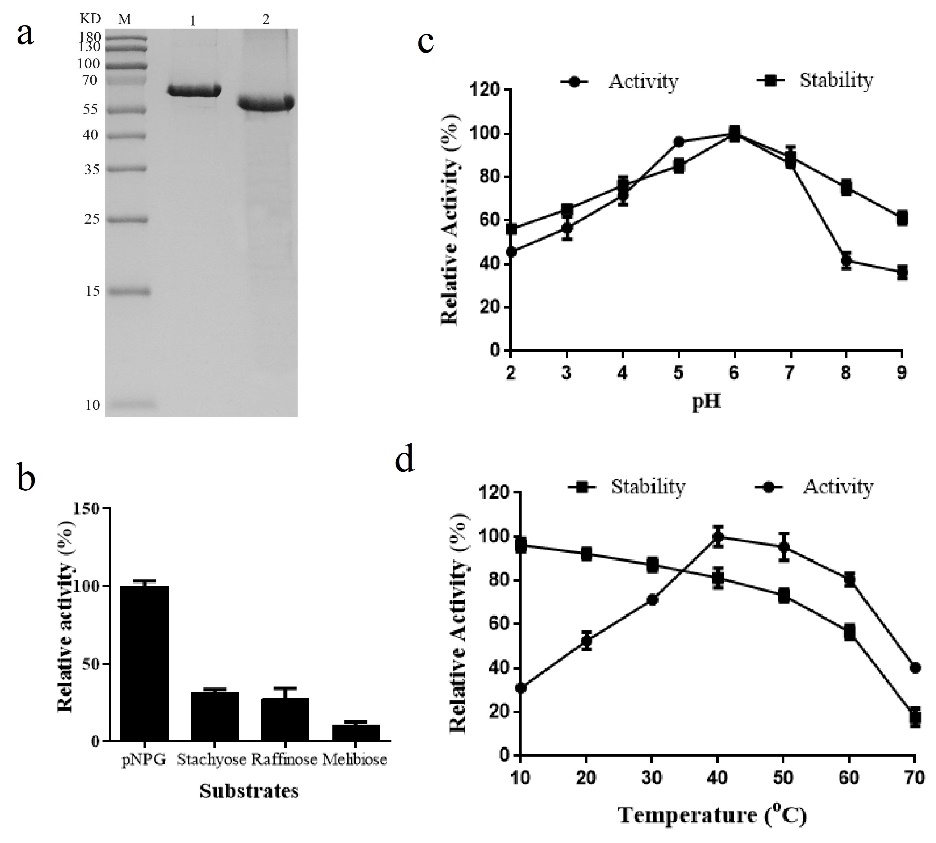


Figure S2. (a) SDS-PAGE analysis of recombinant GalC. Lane M, standard Mw markers; lane 1, purified GalC; lane 2, Bovine albumin. (b) Substrate specificity of GalB (c) The pH activity and stability of GalC. (d) Thetemperature activity and stability of GalC.


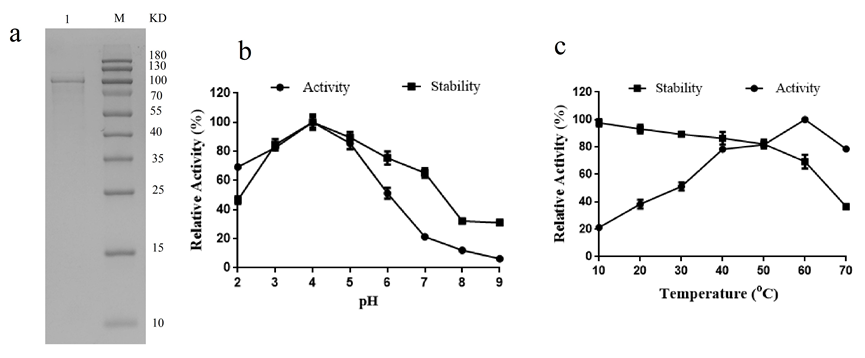


Figure S3. (a) SDS-PAGE analysis of recombinant GalG. Lane M, standard Mw markers; lane 1, purified GalG; lane 2, Bovine albumin. (b) The pH activity and stability of GalG. (c) Thetemperature activity and stability of GalG.

Table S1 Primers for amplifying genes coding galactosidases

| Primers | Sequences |
| --- | --- |
| GA5 | ATGTTTTCTACGAGCCGTTTGTTTC |
| GA3 | CTACACCTTGTACAGCCAGATCAA |
| GB5 | ATGTCTCCAGCATTAGTACTTGC |
| GB3 | TCAACACGAATTCTTGAACAAC |
| GC5 | ATGGGTTTCAACACTTACAACCC |
| GC3 | TCAAGCAACAACACCAATCCTATCG |
| GD5 | ATGACACGCACCACCGAGACCTCT |
| GD3 | CTATTGTCCATAGTAACGCCACTTG |
| GE5 | ATGAGACTCTGCTTGCATCTCCTCG |
| GE3 | CTAAGCATATCGTAGTCTTTCGC |
| GF5 | ATGAAAGGTCTCTTCAAGAACTTG |
| GF3 | CTAGTAGTACTGCAACCTCTCC |
| GH5 | ATGGCTCTGCCCGTAAGCTTGACAG |
| GH3 | CTAGAAATCTATAGCAGGCTCCA |
| GG5 | ATGGTGCTTGGTAAAACTTTCATG |
| GG3 | TTAGTACGCACCCTCTCTCTTCGACC |

Table S2 Primers used to perform qRT-PCR assays

| Primers | Sequences |
| --- | --- |
| AF | AGACTGGACACCAAACCCTG |
| AR | CGGAGATAGCCTTGATGAGA |
| BF | ATGCCGTGACGCACCAGTTA |
| BR | TCTGGCGGGAAGCAGTTGTC |
| CF | CACCCACCGCACCCACTCTT |
| CR | TCACGCTGATGCTCGCTCCC |
| DF | AAGTTCTTATGAGTGCTGGGTA |
| DR | GAGGTACTTGAGCGGTTGAG |
| EF | TCTGGTCGTATCACTCTGCTT |
| ER | CCTTCGGTTATTTGGTTCTT |
| FF | CATCGGCGGTACTGGTAGTGG |
| FR | GGCAAGGGTGGCGTTCAAAA |
| HF | GAGAAATCCAGCCGAGTTTG |
| HR | AGCTTCGCATCTTTGTAGTCA |
| GF | CTCGGCTCCGCTCGTGGTAT |
| GR | GGCCCTCTGGTGCGATCTTC |
| 18SF | AGGTGCAGTTGGTGTTTGGA |
| 18SR | CGCAGCAGCGTTATTCTTTT |
